# Novel classes of non-coding RNAs and cancer

**DOI:** 10.1186/1479-5876-10-103

**Published:** 2012-05-21

**Authors:** Jiri Sana, Petra Faltejskova, Marek Svoboda, Ondrej Slaby

**Affiliations:** 1 Masaryk Memorial Cancer Institute, Department of Comprehensive Cancer Care, Zluty kopec 7, Brno, Czech Republic, Europe; 2 Central European Institute of Technology, Masaryk University, Brno, Czech Republic, Europe; 3 Masaryk Memorial Cancer Institute, Department of Comprehensive Cancer Care, Zluty kopec 7, 656 53, Brno, Czech Republic, Europe

**Keywords:** Non-coding RNAs, microRNAs, siRNAs, piRNAs, lncRNAs, Cancer

## Abstract

For the many years, the central dogma of molecular biology has been that RNA functions mainly as an informational intermediate between a DNA sequence and its encoded protein. But one of the great surprises of modern biology was the discovery that protein-coding genes represent less than 2% of the total genome sequence, and subsequently the fact that at least 90% of the human genome is actively transcribed. Thus, the human transcriptome was found to be more complex than a collection of protein-coding genes and their splice variants. Although initially argued to be spurious transcriptional noise or accumulated evolutionary debris arising from the early assembly of genes and/or the insertion of mobile genetic elements, recent evidence suggests that the non-coding RNAs (ncRNAs) may play major biological roles in cellular development, physiology and pathologies. NcRNAs could be grouped into two major classes based on the transcript size; small ncRNAs and long ncRNAs. Each of these classes can be further divided, whereas novel subclasses are still being discovered and characterized. Although, in the last years, small ncRNAs called microRNAs were studied most frequently with more than ten thousand hits at PubMed database, recently, evidence has begun to accumulate describing the molecular mechanisms by which a wide range of novel RNA species function, providing insight into their functional roles in cellular biology and in human disease. In this review, we summarize newly discovered classes of ncRNAs, and highlight their functioning in cancer biology and potential usage as biomarkers or therapeutic targets.

## Introduction

**Figure 1:**
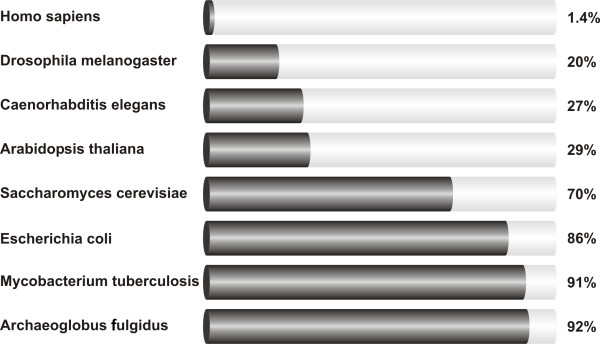
The percentage of protein-coding genes sequences in several eukaryotic and bacterial genomes.

The abundance of non-translated functional RNAs in the cell has been a textbook truth for decades. Most of these non-coding RNAs (ncRNAs) fulfil essential functions, such as ribosomal RNAs (rRNAs) and transfer RNAs (tRNAs) involved in mRNA translation, small nuclear RNAs (snRNAs) involved in splicing and small nucleolar RNAs (snoRNAs) involved in the modification of rRNAs. The central dogma of molecular biology, developed from the study of simple organisms like Escherichia coli, has been that RNA functions mainly as an informational intermediate between a DNA sequence (‘gene’) and its encoded protein. The presumption was that most genetic information that specifies biological form and phenotype is expressed as proteins, which have not only diverse catalytic and structural functions, but also regulate the activity of the system in various ways. This is largely true in prokaryotes and presumed also to be true in eukaryotes [1]. But one of the great surprises of modern biology was definitely the discovery that the human genome encodes only ~20,000 protein-coding genes, representing less than 2% of the total genome sequence (see Figure 1). Subsequently, with the advent of tiling resolution genomic microarrays and whole genome and transcriptome sequencing technologies (ENCODE project) it was determined that at least 90% of the genome is actively transcribed. The human transcriptome was found to be more complex than a collection of protein-coding genes and their splice variants; showing extensive antisense, overlapping and ncRNA expression [1,2]. Although initially argued to be spurious transcriptional noise or accumulated evolutionary debris arising from the early assembly of genes and/or the insertion of mobile genetic elements, recent evidence suggests that the proverbial “dark matter” of the genome may play a major biological role in cellular development, physiology and pathologies. In general, the more complex an organism, the greater is its number of ncRNAs. The enticing possibility that although the number of protein-coding transcripts between organisms is similar, the ultimate control of cellular function may be through interactions between proteins and ncRNA, is corroborated by the fact that the majority of chromatin-modifying complexes do not have DNA binding capacity and therefore, must utilize a third party in binding to DNA. It has been largely demonstrated that this third party may be represented by transcription factors as well as by ncRNAs [2,3] .

The beginnings of the present-day understanding on regulatory non-coding RNAs were inspired mainly by the pioneering ideas of John S. Mattick, who has long argued that proteins comprise only a minority of the eukaryotic genome’s information output. Considering unique ability of RNA to both fold in three-dimensional space and hybridize in a sequence-specific manner to other nucleic acids, ncRNAs are proposed to behave as a digital-to-analogue processing network, allowing the expansion of complexity in biological systems, well beyond purely protein-based regulatory networks [4].

Non-coding RNAs are grouped into two major classes based on transcript size; small ncRNAs and long ncRNAs (lncRNAs) (classification of recently discovered non-coding RNAs is summarized in Table 1). Small ncRNAs are represented by a broad range of known and newly discovered RNA species, with many being associated with 5′ or 3′ regions of protein-coding genes. This class includes the well-documented miRNAs, siRNAs, piRNAs, etc. Most of them significantly extended our view of molecular carcinogenesis, and at present they are subject of intensive translational research in this field. In contrast to miRNAs, lncRNAs are mRNA-like transcripts ranging in length from 200 nt to ~100 kilobases (kb) and lacking significant open reading frames. LncRNAs’ expression levels appear to be lower than protein-coding genes, and some lncRNAs are preferentially expressed in specific tissues. The small number of characterized human lncRNAs have been associated with a spectrum of biological processes including alternative splicing or nuclear import. Moreover they can serve as structural components, precursors to small RNAs and even as regulators of mRNA decay. Furthermore, accumulating reports of misregulated lncRNA (HOTAIR, MALAT1, HULC, T-UCRs, etc.) expressions across numerous cancer types suggest that aberrant lncRNA expression may be an important contributor to tumorigenesis. In this review, we summarize recent knowledge of novel classes of ncRNAs, their biology and function, with special focus on their significance in cancer biology and oncology translational research, which is the field where the number of publications focusing this topic is rapidly growing [5-7].

**Table 1:** Types of recently discovered human non-coding RNAs

	**Class**	**Symbol**	**Characteristic**	**Disease / biological function associations**
**Small non-coding RNAs**	**MicroRNAs**	miRNAs	18–25 nt; account 1–2% of the human genome; control the 50% of protein-coding genes; guide suppression of translation; Drosha and Dicer dependent small ncRNAs	initiation of various disorders including many, if not all, cancers / regulation of proliferation, differentiation, and apoptosis involved in human development
	**Small interfering RNAs**	siRNAs	19–23 nt; made by Dicer processing; guide sequence specific degradation of target mRNA	great potential in diseases treatment / posttranscriptional gene silencing mainly through RISC degradation mechanism; defence against pathogenic nucleic acids
	**Piwi-interacting RNAs**	piRNAs	26–30 nt; bind Piwi proteins; Dicer independent; exist in genome clusters; principally restricted to the germline and somatic cells bordering the germline	relationship between piRNAs and diseases has not yet been discovered / involved in germ cell development, stem self-renewal, and retrotransposon silencing
	**Small nucleolar RNAs**	snoRNAs	60–300 nt; enriched in the nucleolus; in vertebrate are excised from pre-mRNA introns; bind snoRNP proteins	association with development of some cancers / important function in the maturation of other non-coding RNAs, above all, rRNAs and snRNAs; miRNA-like snoRNAs regulate mRNAs
	**Promoter-associated small RNAs**	PASRs	20–200 nt; modified 5′ (capped) ends; coincide with the transcriptional start sites of protein- and non-coding genes; made from transcription of short capped transcripts	relationship with diseases has not yet been discovered / involved in the regulation of the transcription of protein-coding genes by targeting epigenetic silencing complexes
	**Transcription initiation RNAs**	tiRNAs	~ 18 nt ; have the highest density just downstream of transcriptional start sites; show patterns of positional conservation; preferentially located in GC-rich promoters	
	**Centromere repeat associated small interacting RNAs**	crasiRNAs	34–42 nt; processed from long dsRNAs	relationship between crasiRNAs and diseases has not yet been discovered / involved in the recruitment of heterochromatin and/or centromeric proteins
	**Telomere-specific small RNAs**	tel-sRNAs	~ 24 nt; Dicer independent; 2′-O-methylated at the 3′ terminus; evolutionarily conserved from protozoa to mammals; have not been described in human up to now	relationship between tel-sRNAs and diseases has not yet been discovered / epigenetic regulation
	**Pyknons**		subset of patterns of variable length; form mosaics in untranslated and protein-coding regions; more frequently in 3′ UTR	expected association with cancer biology / possible link with posttranscriptional silencing of genes, mainly involved in cell communication, regulation of transcription, signaling, transport, etc.
**Long non-coding RNAs**	**Long intergenic non-coding RNAs**	lincRNAs	ranging from several hundreds to tens of thousands nts; lie within the genomic intervals between two genes; transcriptional cis-regulation of neighbouring genes	involved in tumorigenesis and cancer metastasis / involved in diverse biological processes such as dosage compensation and/or imprinting
	**Long intronic non-coding RNAs**		lie within the introns; evolutionary conserved; tissue and subcellular expression specified	aberrantly expressed in human cancers / possible link with posttranscriptional gene silencing
	**Telomere-associated ncRNAs**	TERRAs	100 bp - >9 kb; conserved among eukaryotes; synthesized from C-rich strand; polyadenylated; form inter-molecular G-quadruplex structure with single-stranded telomeric DNA	possible impact on telomere-associated diseases including many cancers / negative regulation of telomere length and activity through inhibition of telomerase
	**Long non-coding RNAs with dual functions**		both protein-coding and functionally regulatory RNA capacity	deregulation has been described in breast and ovarian tumors / modulate gene expression through diverse mechanisms
	**Pseudogene RNAs**		gene copies that have lost the ability to code for a protein; potential to regulate their protein-coding cousin; made through retrotrans-position; tissue specific	often deregulated during tumorigenesis and cancer progression / regulation of tumor suppressors and oncogenes by acting as microRNA decoys
	**Transcribed-ultraconserved regions**	T-UCRs	longer than 200 bp; absolutely conserved between orthologous regions of human, rat, and mouse; located in both intra- and intergenic regions	expression is often altered in some cancers; possible involvement in tumorigenesis / antisense inhibitors for protein-coding genes or other ncRNAs

### Small non-coding RNAs

**Figure 2:**
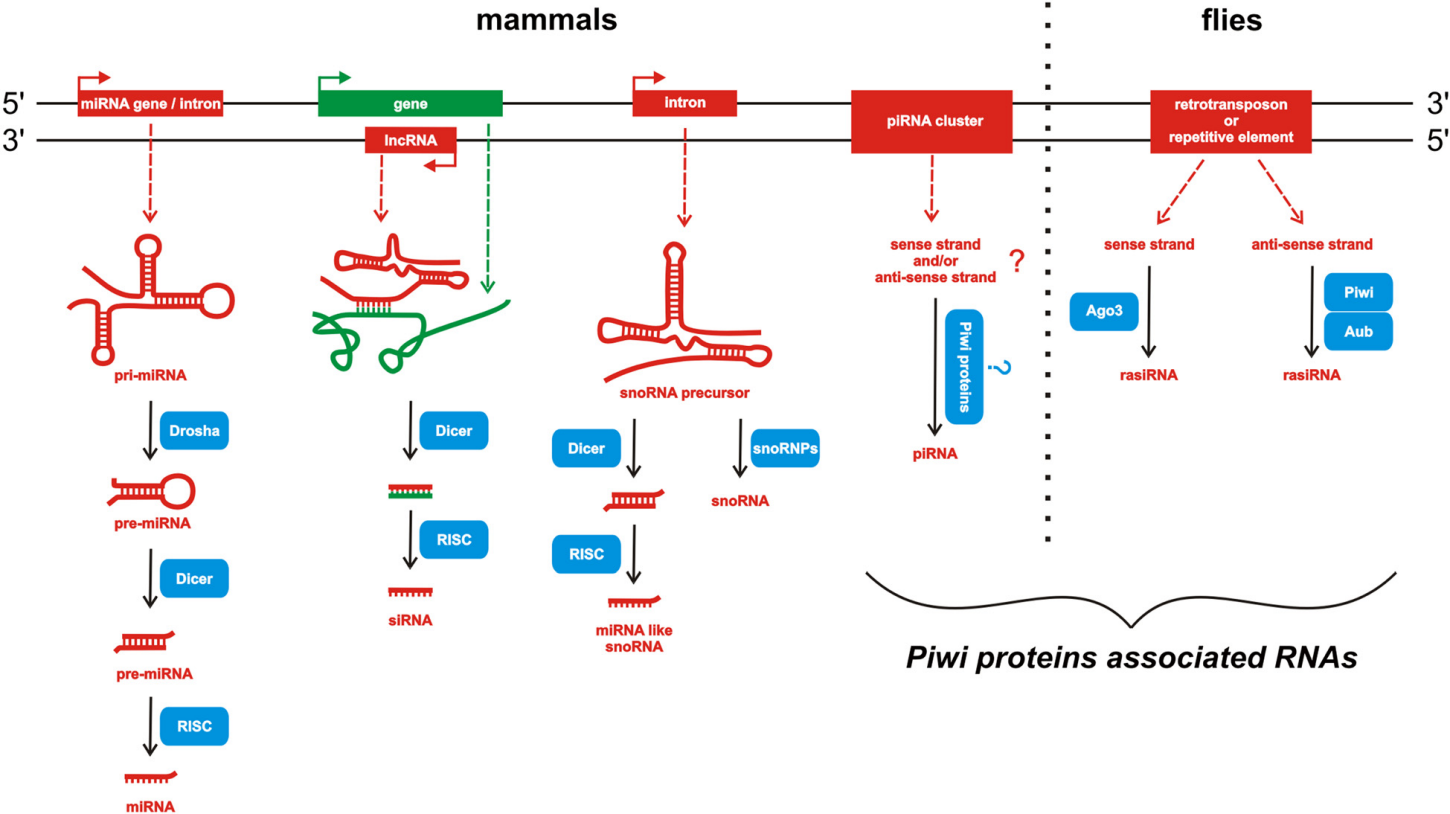
Short ncRNAs biogenesis pathways.

Post-transcriptional RNA silencing or RNA interference (RNAi) is a naturally conserved mechanism of regulation of gene expression described in almost all eukaryotic species including humans [8,9]. It is mostly triggered by dsRNA precursors that vary in length and origin. These dsRNAs are rapidly processed into short RNA duplexes subsequently generating small ncRNAs (small ncRNAs), which are associated with Argonaute family proteins and guide the recognition and ultimately the cleavage or translational repression of complementary single-stranded RNAs, such as messenger RNAs or viral genomic/antigenomic RNAs. Moreover, the small ncRNAs have also been implicated in guiding chromatin modifications [9,10]. Since the discovery of the first small ncRNA, various classes of small ncRNAs have been identified. Based on whether their biogenesis is dependent on Dicer, the dsRNA specific RNA III ribonuclease, all the known eukaryotic small ncRNAs can be classified into two goups: Dicer-dependent, such as microRNAs (miRNAs), small interfering RNAs (siRNAs), and in some cases small nucleolar RNAs (snoRNAs); and Dicer-independent small ncRNAs, such as PIWI-interacting RNAs (piRNAs) [11] (Figure 2). Moreover, phylogenetic analysis indicates that known Argonaute family proteins can be divided into two subgroups namely AGO based on AGO1 and PIWI based on PIWI. Interestingly, Ago proteins interact with miRNAs and siRNAs while Piwi subgroup is characterized by interaction with piRNAs [12]. Biogenesis of other small non-coding RNAs is less or completely undescribed yet. These RNAs are generally classified according to their genome and function localization. Among them belong promoter-associated small RNAs (PASRs), transcription initiation RNAs (tiRNAs), centromere repeat associated small interacting RNAs (crasiRNAs), and telomere-specific small RNAs (tel-sRNAs). To the class of small non-coding RNAs also belong the recently discovered pyknons that, as suggested by current findings, are involved in many biological functions. It was many times described that some of above mentioned small non-coding RNAs play important roles in pathogenesis of various diseases including tumors. In this respect, the most studied ncRNAs are miRNAs, which have been described in many, if not all, cancers [13-16].

### MicroRNAs

The most frequently studied subclass of small ncRNAs are microRNAs (miRNAs), originally discovered by Victor Ambros in *Caenorhabditis elegans*. They are 18–25 nucleotides long, evolutionary conserved, single-stranded RNA molecules involved in specific regulation of gene expression in eukaryotes [17]. It is predicted that miRNA genes account for 1–2% of the human genome and control the activity of ~50% of all protein-coding genes [18,19]. Early annotation for the genomic position of miRNAs indicated that most miRNAs are located in intergenic regions (>1 kb away from annotated or predicted genes), although a sizeable minority was found in the intronic regions of known genes in the sense or antisense orientation. This led to the postulation that most miRNA genes are transcribed as autonomous transcription units [19]. A detailed analysis of miRNA gene expression showed that miRNA genes can be transcribed from their own promoters and that miRNAs are generated by RNA polymerase II (RNAPII) as primary transcripts (pri-miRNAs). These are processed to short 70-nucleotide stem–loop structures known as pre-miRNAs by the ribonuclease called Drosha and the double-stranded-RNA-binding protein known as Pasha (or DGCR8 – DiGeorge critical region 8), which together compose a multiprotein complex termed a microprocessor. The pre-miRNAs are transported to cytoplasm by the RAN GTP-dependent transporter exportin 5 (XPO5). In the cytoplasm, the pre-miRNAs are processed to mature miRNA duplexes by their interaction with the endonuclease enzyme Dicer in complex with dsRNA binding protein TRBP [19,20]. One strand (“guide strand”) of the resulting 18–25-nucleotide mature miRNA duplex ultimately gets integrated into the miRNA-induced silencing complex (miRISC) with the central part formed by proteins of the Argonaute family, whereas the other strand (passenger or miRNA*) is released and degraded. The retained (“guide”) strand is the one that has the less stably base-paired 5′ end in the miRNA/miRNA* duplex. Generally, most miRNA genes produce one dominant miRNA species. However, the ratio of miRNA to miRNA* can vary in different tissues or developmental stages, which probably depends on specific properties of the pre-miRNA or miRNA duplex, or on the activity of different accessory processing factors [19]. Moreover, the ratio might be modulated by the availability of mRNA targets as a result of enhanced destabilization of either miRNA or miRNA* occurring in the absence of respective complementary mRNAs [20]. Mature miRNAs in miRISC exert their regulatory effects by binding to imperfect complementary sites. MiRNAs repress target-gene expression post-transcriptionally, apparently at the level of translation, through a miRISC complex that is similar to, or possibly identical with, that used for the RNAi pathway discussed later. Perfect complementarity of mRNA-miRNA allows Ago-catalyzed cleavage of the mRNA strand, whereas central mismatches exclude cleavage and promote repression of mRNA translation. Consistent with translational control, miRNAs that use this mechanism reduce the protein levels of their target genes, but the mRNA levels of these genes are barely affected [21-23]. Current studies indicate that miRNA targeting in mammalian cells occurs predominantly through binding to sequences within 3′UTRs [24,25], however inhibition of gene expression through targeting the 5′UTR has been also demonstrated [26]. Nevertheless, statistical analyses of conserved miRNA target sequences proved that mammalian miRNA target sites rarely occur within 5′UTRs [24,25,27]. Moreover, it was found out that miR-10a induces, rather than inhibits, protein expression through binding to 5′UTRs of cellular transcripts [23]. It is therefore supposed that binding to 5′UTR results in mechanistic effects divergent from 3′UTR binding.

Most of the miRNAs described to date regulate crucial cell processes such as proliferation, differentiation, and apoptosis. Therefore, these RNAs are involved in human development as well as in initiation of various disorders including many, if not all, cancers where miRNAs have been found to be also significant prognostic and predictive markers [13,28-35]. Examples of miRNAs with significant functional effects in cancer are mentioned below.

Bloomston *et al*. [36] identified 6 miRNAs linked to long-term survival in pancreatic adenocarcinoma. They found also that expression level of miR-196a-2 was able to predict patients’ survival, since higher miRNA levels marked the poor survivors group. In HCC, up-regulation of miR-221 and down-regulation of miR-122 were associated with shorter time to recurrence [37,38]. MiR-21 is up-regulated in many solid tumors, including CRC. Slabý *et al*. [39] proved that miR-21 over-expression shows a strong correlation with the established prognostic factors as nodal stage, metastatic disease and UICC stage. Moreover, Kulda *et al*. [40] correlated miR-21 expression to disease-free interval (DFI). There was shorter DFI in patients with a higher expression of miR-21. Several studies proved that down-regulated expression of miR-221, miR-137, miR-372, miR-182*, let-7 and miR-34a is associated with shorter survival in patients with lung cancer [41-43]. Breast cancer metastatic process has been connected with up-regulation of miR-10b [44] and with loss of expression of miR-126 and miR-335 [45]. Finally, higher levels of miR-15b were associated with poor survival and recurrence in melanoma [46]. Another important question for management of cancer patients is the possibility of predicting therapy response. Nakajima *et al*. [47] identified let-7g and miR-181b as significant indicators for chemoresponse to S-1-based chemotherapy. The same year, Markou *et al*. [48] demonstrated that inhibition of miR-21 and miR-200b increases the sensitivity of cholangiocarcinoma cells to gemcitabine. Yang *et al*. [49] identified miR-214, a miRNA up-regulated in ovarian cancer, as responsible of cisplatin resistance through its action on PTEN/AKT pathway. Subsequently, there is a large number of publications which confirmed many 3′UTRs of oncogenes an tumor suppressor genes to be direct targets of selected miRNAs. According to a recent study by Nagel *et al*. [50], miR-135a and miR-135b decrease translation of the APC transcript *in vitro*. Concerning CRC, KRAS oncogene has been reported to be a direct target of the let-7 miRNA [51]. Another miRNA associated with KRAS regulation is miR-143 [52]. MiRNAs arrays-based studies revealed the p85β regulatory subunit of PI3K as a direct target of miR-126 [53]. Moreover, another important regulatory component of PI3K pathway, the tumor suppressor gene *PTEN*, is strongly repressed by miR-21 in hepatocellular carcinoma [54]. MiR-17-5p belongs to a highly conserved, polycistronic miRNA cluster miR-17-92. Yu *et al*. [55] described the function of this cluster as a negative regulator of cell cycle and proliferation of human breast cancer cells, which directly regulates cyclin D1 (CCND1). The same cluster is also involved in malignancies of B cell origin [56] and a direct regulation by c-MYC has been reported [57,58]. Some of the most often deregulated miRNAs with their experimentally proved mRNA targets are summarized in the Table 2, however, the number of described miRNAs and putative targets is much more higher and it is not possible to mention all of them.

**Table 2:** Gene targets of the most common described human cancer-associated miRNAs

**MiRNA**	**Associated cancers**	***In vitro*confirmed gene targets**
**MiR-21**	CRC, PC, RCC, GBM, BrC, NSCLC, BCL, PTC, HCC, HNSCC, ESCC, GC, CML, CCC, MM, OC, M, LC, PDA	PDCD4, TIMP3, RhoB, Spry1, PTEN, TM1, CDK2AP1, ANP32A, SMARCA4, ANKRD46, THRB, Cdc25A, BMPRRII, LRRFIP1, BTG2, MARCKS, TPM1
**MiR-155**	NSCLC, SCLC, HCC, BrC, M, CCC, HL, PDA, RCC, GBM, PTC, CML, CRC, SPA, AML, NPC, CLL	FOXO3A, SOX6, SATB1, SKI, Wee1, SOCS1, SHIP1, S/EBPβ, IFN-γRα, AGTR1, FGF7, ZNF537, ZIC3, IKBKE, RhoA, BACH1, ZIC3, HIVEP2, CEBPB, ZNF652, ARID2, SMAD5, TP53INP1
**MiR-145**	BrC, CRC, ESCC, NSCLC, PC, BCL, OC, GC, BlC, NPC, HCC	c-Myc, ERK5, FSCN1, SMAD2/3, IGF-1R, FLI1, DFF45, mucin 1, MYO6, CBFB, PPP3CA, CLINT1, ICP4, RTKN
**MiR-221**	BrC, PC, CRC, M, GBM, ALL, HCC, PTC, PDA, GC, CML, NSCLC, AML, OC	DVL2, KIT, CDKN1B, Bmf, p27, HOXB5, CDKN1C/p57, CDKN1B/p27, MMP1, SOD2, TIMP3, Dicer1, ERα, ARHI, PUMA, p27Kip1, p57
**MiR-222**		
**Let-7a**	M, HL, nHL, CRC, SLC, NSCLC, GC, HNSCC, ESCC, OC, CLL, HCC	PRDM-1, STAT3, Caspase-3, Integrin β3, PRDM1/blimp-1
**MiR-16**	LC, OC, NPC, GC, PC, BrC, HCC, MM, CLL, HL	VEGFR2, FGFR1, Zyxin, Cyclin E1, Bmi-1, BRCA-1, BCL2
**MiR-200**	BrC, PDA, GC, HNSCC, M, OC, PC	FN1, MSN, NTRK2, LEPR, ARHGAP19, ZEB1/2, Flt1/VEGFR1, FAP-1, FOG2, ERRFI-1
**MiR-205**	M, BrC, PC, ESCC, HNSCC	Runx2, E2F1, ErbB3, Zeb1
**MiR-31**	PTC, CRC, BrC, LC, GC, HCC	LATS2, WAVE3, SATB2, ITGA5, RDX, RhoA, FIH
**MiR-126**	CRC, GC, BrC, SCLC, AML, NSCLC, HCC	SLC7A5, SOX2, PLAC1, VEGFA, PIK3R2, Crk, EGFL7, p85beta
**MiR-210**	PDA, RCC, BrC, PC, GBM, NSCLC, OC, GC, HNSCC	FGFRL1, SDHD, MNT
**MiR-9**	GBM, PC, nHL, EC, OC	CAMTA1, PDGFR-β, CDX2, PRDM-1, E-cadherin, NF-kappaB1
**MiR-141**	PC, EC, CRC, HNSCC, LC, BrC, ESCC, OC, RCC	SIP1, YAP1
**MiR-122**	HCC, RCC	Bcl-w, ADAM17

CRC colorectal cancer, PC prostate cancer, RCC renal cell carcinoma, GBM glioblastoma multiforme, BrC breast cancer, LC lung cancer, NSCLC non-small cell lung cancer, SCLC small cell lung cancer, BCL B-cell lymphoma, PTC papillary thyroid carcinoma, HCC hepatocellular carcinoma, HNSCC head and neck squamous cell carcinoma, ESCC esophagus squamous cell carcinoma, GC gastric cancer, CLL chronic lymphocytic leukemia, CML chronic myelogenous leukemia, ALL acute lymphocytic leukemia, AML acute myeloid leukemia, CCC cervical cell carcinoma, MM multiple myeloma, OC ovarian cancer, M melanoma, LC laryngeal carcinoma, PDA pancreatic ductal adenocarcinoma, HL Hodgkin lymphoma, nHL Non-Hodgkin lymphoma, SPA sporadic pituitary adenomas, NPC nasopharyngeal carcinoma, BlC bladder cancer, EC endometrial cancer.

### Small interfering RNAs

Another class of small ncRNAs involved in post-transcriptional RNA silencing are so-called small interfering RNAs (siRNAs). They are produced from long dsRNAs of exogenous or endogenous origin [59]. These short helical RNA molecules are formed by two at least partially complementary RNA single strands, namely the passenger strand and the guide strand. Typical strand lengths of these dsRNAs are 19–23 nucleotides and they are made by Dicer processing as miRNAs [60]. One of the arisen single strands is subsequently incorporated into RISC (RNA-induced silencing complex) where guides sequence-specific degradation of complementary target mRNAs unlike miRNA that rather suppresses translation and does not lead to degradation of the mRNA target [9,61,62]. SiRNAs are worldwide used in gene silencing experiments and have become a specific and powerful tool to turn off the expression of target genes, and also turned into a promising experimental tool in molecular oncology. SiRNAs could be used in cancer therapy by several strategies. These include the suppression of overexpressed oncogenes, retarding cell division by interfering with cyclins and related genes or enhancing apoptosis by inhibiting anti-apoptotic genes. For example, Vassilev *et al*. [63] developed new siRNA-based inhibitors of the p53-MDM2 protein interaction. A year later, Wu *et al*. [30] demonstrated that down-regulation of RPL6 (ribosomal protein L6) in gastric cancer SGC7901 and AGS cell lines by siRNA reduced colony forming ability and cell growth. Moreover, the cell cycle of these cells was suppressed in G1 phase. Similarly, CDK8 specific siRNA transfection down-regulated the expression of CDK8 in colon cancer cells, which was also associated with a decrease in the expression of β-catenin, inhibition of proliferation, increased apoptosis and G0/G1 cell cycle arrest [64]. Dufort *et al*. [65] described that cell transfection of IGF-IR siRNAs decreased proliferation, diminished phosphorylation of downstream signaling pathway proteins, AKT and ERK, and caused a G0/G1 cell cycle block in two murine breast cancer cell lines, EMT6 and C4HD. The IGF-IR silencing also induced secretion of two proinflammatory cytokines, TNF-α and IFN-γ. Another study showed that mTOR-siRNA transfection significantly inhibits cell proliferation, increases the level of apoptosis and decreases migration of NSCLC cells, and could be used as an alternative therapy targeting mTOR with fewer side effects [66]. RNAi against multidrug resistance genes or chemoradioresistance and angiogenesis targets may also provide beneficial cancer treatments. He *et al*. [67] proved that silencing of MDR1 by siRNA led to decreased P-glycoprotein activity and lower drug resistance of L2-RAC cells, which could be used as a novel approach of combined gene and chemotherapy for yolk sac carcinoma. Another study showed that combination of proteasome inhibitors with Mcl-1 siRNA enhances the ultimate anticancer effect in DLD-1, LOVO, SW620, HCT-116, SKOV3 and H1299 cell lines [68]. Bansal *et al*. [69] states that selective siRNA depletion of CDK1 increases sensitivity of patients with ovarian cancer to cisplatin-induced apoptosis. The number of publications dealing with siRNAs is rapidly growing and successful cancer therapy by siRNA *in vitro* and *in vivo* provides the enthusiasm for potential therapeutic applications of this technique [70]. Some examples of siRNA cancer therapies in clinical trials are summarized in Table 3.

**Table 3:** **Small RNA-based therapeutics in clinical trials (adapted from**[71]**)**

**Gene target**	**Drug type**	**Drug name**	**Clinical phase**	**Notes**
Bcl-2	LNA-oligo	SPC2996	I/II	CLL
Immunoproteasome β-subunits LMP2, LMP7 and MECL1	siRNA	Proteasome siRNA	I	Metastatic lymphoma
PLK1	siRNA	PLK SNALP	pre-clinical	
M2 subunit of ribonucleotide reductase	siRNA	CALAA-01	I	Solid tumors
PKN3	siRNA	Atu027	I	Solid tumors
KSP and VEGF	siRNA	ALN-VSP	I	Solid tumors
Survivin	LNA-oligo	EZN3042	I/II	Solid tumors
HIF-1α	LNA-oligo	EZN2968	I/II	Solid tumors
Furin	shRNA	FANG vaccine	I	Solid tumors
eiF-4E	LNA-oligo	elF-4E ASO	I	Solid tumors
Survivin	LNA-oligo	Survivin ASO	II	Solid tumors

### Piwi proteins associated RNAs

Extensive research in the past few years has revealed that members of the Argonaute protein family are key players in gene-silencing pathways guided by small RNAs. This family is further divided into AGO and PIWI subfamilies [72]. It was proved that the AGO proteins are present in diverse tissues and bind to miRNAs and siRNAs, whereas PIWI proteins are especially present in germline, and associate with a new class of small ncRNAs termed PIWI-interaction RNAs (piRNAs). PiRNAs are typically 24–32 nucleotides long RNAs that are generated by a Dicer-independent mechanism. It was thought that they are derived only from transposons and other repeated sequence elements [73] and therefore, they were alternatively designated as repeat-associated small interfering RNAs (rasiRNAs) [74]. But it is now clear that piRNAs can be also derived from complex DNA sequence elements [75] and that rasiRNAs are a subset of piRNAs.

The precise mechanism of piRNAs biogenesis is not clear, but in 2007 Brennecke *et al*. [73] described a new mechanism similar to secondary siRNA generation, called as ping-pong model. He observed that antisense piRNAs associate with PIWI/AUB complex while sense piRNAs associate with AGO3 protein. This information led to the suggestion that PIWI and AUB proteins bind to maternally deposited piRNAs (primary piRNA) and this complex is subsequently bound to the transcripts produced by retrotransposons and cleaves a transcript generating a sense piRNAs (secondary piRNAs) that bind to AGO3. Finally, piRNA-AGO3 complex binds to the retrotransposon transcript, creating another set of anti-sense piRNAs. However, the model of piRNAs biogenesis is still incomplete and precise mechanisms of action remain poorly characterized (for a review, see [76-78]).

The PIWI subfamily as well as piRNAs have been implicated in germ cell development, stem cell self-renewal, and retrotransposon silencing. Recently, several studies were published describing the association between HIWI (the human ortholog of PIWI) expression and diverse group of cancers including pancreatic [79] and gastric [80] adenocarcinomas, sarcomas [81], hepatocellular carcinomas [82], colorectal cancer [83], gliomas [84] and esophageal squamous cell carcinomas [85]. It was proved that higher levels of HIWI mRNA are connected with worse clinical outcome. Moreover, the expression patern of HIWI in gastric cancer tissues was similar to that of Ki67 and suppression of HIWI induced cell cycle arrest in G2/M phase [80]. Lee *et al*. [86] described that PIWIL2 (PIWI-like 2) protein is widely expressed in tumors and inhibits apoptosis through activation of STAT3/BCL-X(L) signalling pathway. Similarly, the newest study of Lu *et al*. [87] shows that this protein forms a PIWIL2/STAT3/c-Src complex, where STAT3 is phosphorylated by c-Src and translocated to nucleus. Subsequently, STAT3 binds to *P53* promoter and represses its transcription. These findings indicate that PIWI proteins may be involved in the development of different types of cancer and could be a potential target for cancer therapy. Recently, it was also proved, that not only PIWI proteins, but also piRNAs can play an important role in carcinogenesis. It was discovered that expression of piR-823 in gastric cancer tissues was significantly lower than in non-cancerous tissues. Artificial increase of the piR-823 levels in gastric cancer cells inhibited their growth. Moreover, the observations from the xenograft nude mice model confirmed its tumor suppressive properties [88]. On the contrary, levels of the piR-651 were upregulated in gastric, colon, lung, and breast cancer tissues compared to the paired non-cancerous tissues. The growth of gastric cancer cells was efficiently inhibited by a piR-651 inhibitor and the cells were arrested at the G2/M phase [89]. Interestingly, the peripheral blood levels of piR-651 and piR-823 in the patients with gastric cancer were significantly lower than those from controls. Thus, piRNAs may be valuable biomarkers for detecting circulating gastric cancer cells [90]. Resolving the function of PIWI proteins and piRNAs has broad implications not only in understanding their essential role in fertility, germline, stem cell development, and basic control and evolution of animal genomes, but also in the biology of cancers [12].

### Small nucleolar RNAs

Small nucleolar RNAs (snoRNAs), 60 – 300 nucleotides long, represent one of the abundant groups of small ncRNAs characterized in eukaryotes. SnoRNAs are enriched in the nucleolus, which is the most prominent organelle in the interphase nucleus providing the cellular locale for the synthesis and processing of cytoplasmic ribosomal RNAs (rRNAs) [91]. Most of the snoRNAs are located within introns of protein-coding genes and are transcribed by RNA polymerase II, however, they can also be processed from introns of longer ncRNA precursors [92]. Nevertheless, while vertebrate snoRNAs are prevalently excised from pre-mRNA introns, in plant and yeast these RNAs are mainly generated from independent transcription units, as either monocistronic or (especially in plants) polycistronic snoRNA transcripts [93].

All snoRNAs fall into two major classes based on the presence of short consensus sequence motifs. First group contains the box C (RUGAUGA) and D (CUGA) motifs, whereas members of the second group are characterized by the box H (ANANNA) and ACA elements [94]. In both classes of snoRNAs, short stems bring the conserved boxes close to one another to constitute the structural core motifs of the snoRNAs, which coordinate the binding of specific proteins to form small nucleolar RNPs (snoRNPs) distinct for both groups [91,95]. SnoRNAs have important functions in the maturation of other non-coding RNAs. Above all, they manage post-transcriptional modification of rRNA and snRNA by 2′-O-methylation and pseudouridylation (for a review, see [91]). Interestingly, it was identified number of human snoRNAs with miRNA-like function. These snoRNAs are processed to small 20–25 nucleotides long RNAs that stably associate with Ago proteins. Processing is independent of the Drosha, but requires Dicer. Moreover, cellular target mRNA, whose activity is regulated by snoRNA, was identified [96].

Several studies have indicated that alterations of snoRNAs play important functions in cancer development and progression. The first report linking snoRNAs to cancer was published in 2002 by Chang *et al.*[97]. He proved that h5sn2, a box H/ACA snoRNA, was significantly downregulated in human meningiomas compared with normal brain tissues. Subsequently, Dong *et al*. [98] identified snoRNA U50 as a reasonable candidate for the 6q tumor-suppressor gene in prostate cancer and this statement was confirmed in another study describing involvement of snoRNAs U50 in the development and/or progression of breast cancer [99]. Interestingly, chromosome 6q14-15 is a breakpoint of chromosomal translocation t(3;6)(q27;q15) for human B-cell lymphoma [100]. The same year, the GAS5 (growth arrest-specific transcript 5) was identified to control mammalian apoptosis and cell growth. GAS5 transcript levels were found to be significantly lower in breast cancer samples relative to adjacent unaffected normal breast epithelial tissues and despite the fact that this gene has no significant protein-coding potential, it was proved that several snoRNAs are encoded in its introns [101]. By profiling ncRNAs signatures in NSCLC tissues and matched noncancerous lung tissues, four snoRNAs (snoRD33, snoRD66, snoRD76 [102] and snoRA42 [103]) were found to be overexpressed in lung tumor tissues and it is supposed that they could be used as potential markers for early detection of non-smal cell lung cancer [102]. Moreover, snoRD33 is located at chromosome 19q13.3 that contains oncogenes involved in different malignances including lung cancer, whereas snoRD66 and snoRD76 are located at chromosomal regions 3q27.1 and 1q25.1, respectively. These two chromosomal segments are the most frequently amplified in human solid tumors [28,104,105]. Recently, low levels of four snoRNAs (RNU44, RNU48, RNU43, RNU6B), commonly used for normalization of miRNA expression, were associated with a poor prognosis of the cancer patients [106]. Martens-Uzunova *et al*. [107]analyzed the composition of the entire small transcriptome by Illumina/Solexa deep sequencing and he revealed several snoRNAs with deregulated expression in samples of patients with prostate cancer. The newest publication concerning snoRNAs proved that snoRD112-114 located at the DLK1-DIO3 locus are ectopically expressed in acute promyelotic leukemia (APL), which shows that a relationship exists between a chromosomal translocation and expression of snoRNA loci. Moreover, *in vitro* experiments revealed that the snoRD114-1 [14q(II-1)] variant promotes cell growth through G0/G1 to S phase transition mediated by the Rb/p16 pathways [108]. Finally, it was also published that snoRNAs are present in stable form in plasma and serum samples [102,106] and therefore could be used as fluid-based biomarkers for cancers. These facts indicate that snoRNAs are critically associated with the development and progression of cancer, however further research for comprehensive understanding their role in carcinogenesis is required.

### Promoter-associated RNAs

Recently, a new class of ncRNAs known as promoter-associated RNAs (paRNAs) (sometimes termed as promoter-upstream transcripts – PROMPTs [109], transcription start site-associated RNAs [110] or promoter-proximal transcription start site RNAs [111]), were discovered. These ncRNAs are derived from eukaryotic promoters and have the potential to regulate the transcription of protein-coding genes by targeting epigenetic silencing complexes [71,112,113]. Their size ranged from 18 to 200 nucleotides and they include long, small and tiny RNAs.

The short paRNAs (PASRs) were identified in 2007 [114] using RNA maps. They are located near the promoter or transcription start site (TSS), but they are not associated with a known protein-coding genes. These transcripts are 20–90 nt long and it was proved that they are not Dicer product [110]. Human PASRs are expressed at low levels and their number per gene is positively correlated with promoter activity and mRNA level [109]. The tiny paRNAs or transcription iniciation RNAs (tiRNAs) are shorter than 23 nt and they are transcribed in both sense and antisense directions around the promoter [115]. Furthermore, they are closely associated with highly expressed promoters and are preferentially located in GC-rich promoters [71,115]. It is still unclear how these two classes of small RNAs are related to one another, or if they share common biogenesis pathways [115]. Recently, a long paRNAs (PALRs, 100–200 nt) has been identified at a single-gene level and they were associated with regulatory functions (for a review, see [112,113,116,117]), especially with modification of DNA methylation [118].

It is supposed, that because of potential of paRNAs to regulate transcription, their deregulation could be associated with different types of diseases, including cancer. It was proved, that transfection of mimetic paRNAs into HeLa and HepG2 cells resulted in the transcriptional repression of human C-MYC and connective tissue growth factor (CTGF) [119]. Hawkins *et al*. [120] described that targeting of the human ubiquitin C gene (*UbC*) with a small paRNA led to long-term silencing which correlated with an early increase in histone methylation and a later increase in DNA methylation at the targeted locus. Furthermore, it was shown that PASRs play an important role in maintaining accessible chromatin architecture for transcription and releasing negative supercoils during transcription [110]. Concerning tiRNAs, they may have similar functions like PASRs, moreover they are usually found at CTCF-binding sites. Taft *et al*. [121] proved, that overexpression of tiRNAs decreased CTCF binding and associated gene expression, whereas inhibition of tiRNAs resulted in increased CTCF localization and associated gene expression. Wang *et al*. [122] described, that an RNA-binding protein TLS (for translocated in liposarcoma) can specifically bind to CREB-binding protein (CBP) and p300 histone acetyltransferase depending on its allosteric modulation by PALRs, and so repress gene target CCND1 in human cell lines. Finally, it was shown that paRNAs have the potential to form double-stranded RNAs and to be processed into endogenous siRNAs [123]. These facts indicate, that this novel class of ncRNAs has a great potential to regulate expression of various tumor suppressors and oncogenes on transcriptional level and therefore be involved in human cancerogenesis.

### Centromere repeat associated small interacting RNAs

Cell stresses can induce incorrect centromere function manifesting in loss of sister chromatid cohesion, abnormal chromosome segregation, and aneuploidy, which have been observed in many human diseases including cancers [124]. These defects are often correlated with the aberrant accumulation of centromere satellite transcripts [125]. Morover, it was observed that human cells under stress accumulate large transcripts of SatIII satellites [126]. The accumulation of similar transcripts in vertebrate cells is thought to result from defective RNA processing of larger transcripts that leads to a reduction of the small RNAs that participate in the recruitment of specific histones critical for centromere function [125,127]. The research on mammalian model uncovered the strong bidirectional promoter capability of the kangaroo endogenous retrovirus (KERV-1) LTR to produce long double-stranded RNAs for both KERV-1 and surrounding sequences, including sat23. These long dsRNAs are then processed into centromere repeat associated small interacting RNAs (crasiRNAs), 34 - 42 nucleotides in length. Unfortunately, the mechanism by which full-length KERV-1 and sat23 transcripts are processed into crasiRNAs remains unknown. The crasiRNAs are involved in the recruitment of heterochromatin and/or centromeric proteins. These findings have profound implications for understanding of centromere function and epigenetic identity by suggesting that a retrovirus, KERV-1, may participate in the organization of centromere chromatin structures indispensable to chromosome segregation in vertebrates [124]. These small centromere-associated ncRNAs occur conserved among eukaryotes suggesting their impact also in human.

### Telomere-specific small RNAs

Another group of recently described short ncRNAs are telomere-specific small RNAs (tel-sRNAs). Tel-sRNAs are ~ 24 nt long, Dicer-independent, and 2′-O-methylated at the 3′ terminus. They are asymmetric with specificity toward telomere G-rich strand, and evolutionarily conserved from protozoan to mammalian cells. Interestingly, tel-sRNAs are up-regulated in cells that carry null mutation of H3K4 methyltransferase MLL and down-regulated in cells that carry null mutations of histone H3K9 methyltransferase SUV39H, suggesting that they are subject to epigenetic regulation. These results support that tel-sRNAs are heterochromatin associated pi-like small RNAs [128]. Recently, it was also reported that an 18-mer RNA oligo of (UUAGGG)_3_ has potential to inhibit telomerase TERT activity *in vitro* by RNA duplex formation in the template region of the telomerase RNA component [129]. Therefore, it is supposed that tel-sRNAs containing UUAGGG repeats could act as sensors of chromatin status and create a feedback loop between the telomeric heterochromatic regulation and telomere length control. Although tel-sRNAs have not been described in human until to date, they could play an important role in carcinogenesis and contribute to unlimited replicative potencial of cancer cells.

### Pyknons

Pyknons are a subset of 127998 patterns of variable length, which form mosaics in untranslated as well as protein-coding regions of human genes. Nevertheless, they are found more frequently in the 3′UTR of genes than in other regions of the human genome [130,131]. Pyknons are present in statistically significant manner in genes that are involved in specific processes such as cell communication, transcription, regulation of transcription, signaling, transport, etc. Pyknons involve ~ 40% of the known miRNA sequences, thus suggesting possible link with posttranscriptional gene silencing and RNA interference [131]. Different sets of pyknons are connected to allele-specific sequence variations of disease-associated SNPs and miRNAs, suggesting that increased susceptibility to multiple common human disorders is associated with global alterations in genome-wide regulatory templates affecting the biogenesis and functions of non-coding RNAs [132].

In the time since their discovery, evidence has been slowly accumulating that these pyknon motifs mark transcribed, non-coding RNA sequences with potential functional relevance in human disease. Tsirigos *et al*. [133] described two GO terms (GO:0006281/DNA repair, GO:0006298/mismatch repair) that were significantly enriched in pyknons-containing regions of the human introns. He pointed out that these two terms are uniquely associated with pyknons and a search of the ENSEMBL database [134] for human genes labeled with these two GO terms identified a *MLH1* gene, that has been associated with hereditary non-polyposis colorectal cancer and other types of carcinomas and microsatellite instabilities. The human MLH1 transcript has 17 introns and the authors proved that these introns contain more than 10 different pyknons. Nevertheless, further research for comprehensive understanding their role in carcinogenesis is necessary.

### Long non-coding RNAs

**Figure 3:**
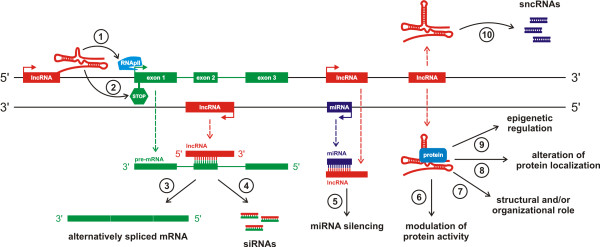
**Schematic illustration of lncRNAs functioning.** LncRNA transcribed from an upstream non-coding promoter can negatively **(1)** or positively **(2)** affect expression of the downstream gene by inhibiting RNA polymerase II recruitment and/or inducing chromatin remodeling, respectively. LncRNA is able to hybridize to the pre-mRNA and block recognition of the splice sites by the spliceosome, thus resulting in an alternatively spliced transcript **(3)**. Alternatively, hybridization of the sense and antisense transcripts can allow Dicer to generate endogenous siRNAs **(4)**. The binding of lncRNA to the miRNA results in the miRNA function silencing **(5)**. The complex of lncRNA and specific protein partners can modulate the activity of the protein **(6)**, is involved in structural and organization roles of the cell **(7)**, alters the protein localizes in the cell **(8)**, and affects epigenetic processes **(9)**. Finally, long ncRNAs can be processed to the small RNAs **(10)**.

**Figure 4:**
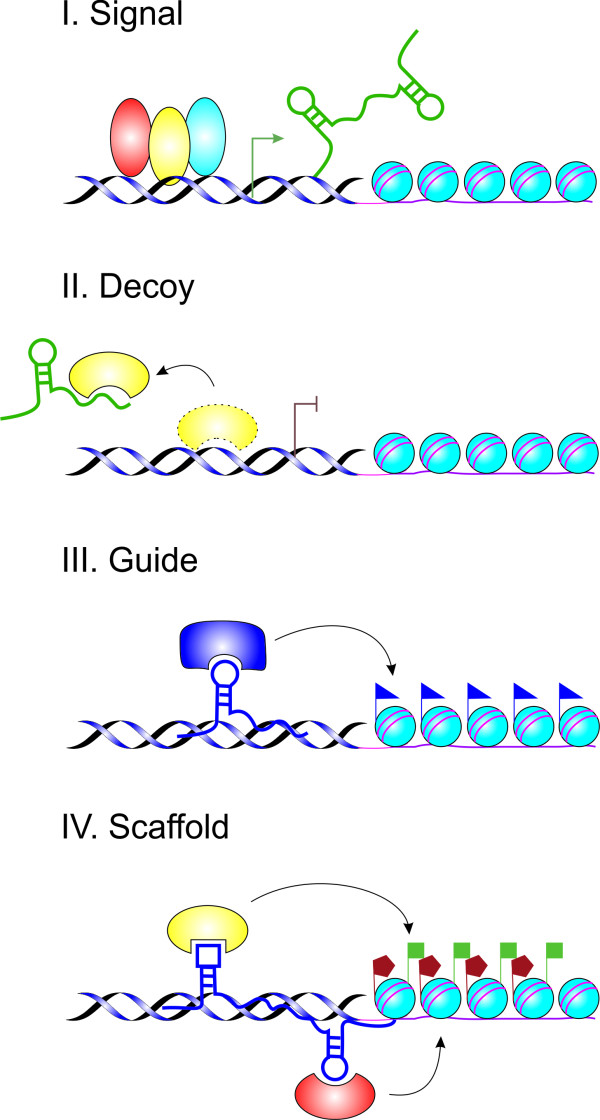
**Schematic diagram of the four mechanisms of lncRNAs functioning. A**, lncRNAs can function as signals and regulate gene expression. **B**, lncRNAs can titrate transcription factors and other proteins away from chromatin or they can function as decoy for miRNA target sites. **C**, lncRNAs can recruit chromatin-modifying enzymes to target genes and therefore function as guides. **D**, lncRNAs can bring together multiple proteins to form ribonucleoprotein complexes (modified according to [143]).

Long non-coding RNAs (lncRNAs) are the broadest class encompassed all non-protein-coding RNA species with length more than 200 nucleotides, however, frequently ranging up to 100 kb. Many identified lncRNA are transcribed by RNA polymerase II (RNAPII), spliced, and usually contain canonical polyadenylation signals, but this is not a fast rule [2]. On the other hand, Pagano *et al*. [135] found out that some of these lncRNAs are due to their promoter structure likely to be transcribed by polymerase III (RNAPIII) and he marked them as cogenes since they could specifically coact with a protein-coding pol II gene. There is substantial evidence to suggest that lncRNAs mirror protein coding genes. Additionally, lncRNAs’ promoters are bound and regulated by transcriptional factors and epigenetically marked with specific histone modifications [136]. LncRNAs are developmentally and tissue specific, and have been associated with a spectrum of biological processes, for example, alternative splicing, modulation of protein activity, alternation of protein localization, and epigenetic regulation. LncRNAs can be also precursors of small RNAs and even tools for miRNAs silencing [71,137-141]. However, one of their primary tasks appears to be regulators of protein-coding gene expression (Figure 3) [142]. Recently, Wang *et al*. [143] described four different mechanisms of lncRNAs action. He supposes that these molecules can function as signals, decoys, guides or as scaffolds (Figure 4). It is not surprising, then, that dysregulation of lncRNAs seems to be an important feature of many complex human diseases, including cancer (Table 4), ischaemic heart disease [144] and Alzheimer’s disease [145]. Also dysregulation of lncRNAs that function as regulators of the expression of tumor suppressors or oncogenes, and not the protein-coding sequence itself, may be one of the ‘hits’ that leads to oncogenesis [2]. That is why they might be suitable as potential biomarkers and targets for novel therapeutic approaches in the future.

**Table 4:** **Human cancer associated lncRNAs (adapted from**[4]**)**

**LncRNA**	**Size**	**Cytoband**	**Cancer types**	**References**
**HOTAIR**	2158 nt	12q13.13	breast	[7,140]
**MALAT1/α/NEAT2**	7.5 kb	11q13.1	breast, lung, uterus, pancreas, colon, prostate, liver, osteosarcoma, neuroblastoma, cervix	[146-151]
**HULC**	500 nt	6p24.3	liver	[152,153]
**BC200**	200 nt	2p21	breast, cervix, esophagus, lung, ovary, parotid, tongue	[154,155]
**H19**	2.3 kb	11p15.5	bladder, lung, liver, breast, endometrial, cervix esophagus, ovary, prostate, colorectal	[156-159]
**BIC/MIRHG155/MIRHG2**	1.6 kb	21q11.2	B-cell lymphoma	[160]
**PRNCR1**	13 kb	8q24.2	prostate	[161]
**LOC285194**	2105 nt	3q13.31	osteosarcoma	[162]
**PCGEM1**	1643 nt	2 g32.2	prostate	[163-165]
**UCA1/CUDR**	1.4–2.7 kb	19p13.12	bladder, colon, cervix, lung, thyroid, liver, breast, esophagus, stomach	[166]
**DD3/PCA3**	0.6–4 kb	9q21.22	prostate	[167,168]
**anti-NOS2A**	1.9 kb	17q23.2	brain	[169]
**uc.73A**	201 nt	2q22.3	colon	[170]
**uc.338**	590 nt	12q13.13	liver	[171]
**ANRIL/p15AS/CDK2BAS**	34.8 kb	9p21.3	prostate, leukemia	[172-175]
**MEG3**	1.6 kb	14q32.2	brain	[176-178]
**GAS5/SNHG2**	isoforms	1q25.1	breast	[101]
**SRA-1/SRA**	1965 nt	5q31.3	breast, uterus, ovary	[179,180]
**PTENP1**	3.9 kb	9p13.3	prostate	[181,182]
**ncRAN**	2186 nt 2087 nt	17q25.1	bladder, neuroblastoma	[183,184]
**LSINCT5**	2.6 kb	5p15.33	breast, ovary	[185]

### Long intergenic non-coding RNAs

Long intergenic non-coding RNAs (lincRNAs) are newly discovered ncRNAs belonging to lncRNAs. RNAs of this subclass ranging in length from several hundred to tens of thousands of bases and they lie within the genomic intervals between two genes. More than 3000 human lincRNAs have been identified, but less than 1% has been characterized [136,186]. It was shown that distinct lincRNAs are involved in diverse biological processes such as imprinting or cancer metastasis [7,140,186]. Moreover, recent studies proved that lincRNAs are exquisitely regulated during development and in response to diverse signaling cues, and exhibit distinct gene expression patterns in primary tumors and metastases [136]. Therefore, these lncRNAs could be utilized for cancer diagnosis, prognosis, and serve as potential therapeutic targets.

**Figure 5:**
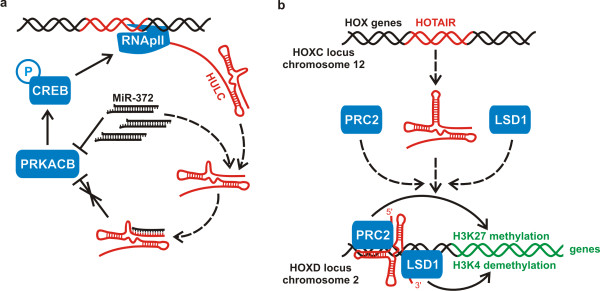
Proposed mechanism of HULC up-regulation in hepatocellular carcinoma (a) and HOTAIR mediated gene silencing of 40 kb of the HOXD locus (b).

Recently it has been demonstrated that lncRNAs can act as natural ‘miRNA sponges’ to reduce miRNA levels [155]. The most highly upregulated transcript found in a microarray-based study of gene expression in hepatocellular carcinoma was determined to be the ncRNA HULC, or Highly Upregulated in Liver Cancer. Transcribed from chromosome 6p24.3, this lncRNA demonstrates the hallmarks of a typical mRNA molecule, including a single spliced GT-AG intron, canonical polyadenylation signals upstream of the poly(A) tail and nuclear export demonstrating strong localization to the cytoplasm. Although HULC was found to co-purify with ribosomes, no translation product for this lncRNA has been detected, supporting its classification as a non-coding transcript [156]. In addition to liver cancer, HULC was found to be highly upregulated in hepatic colorectal cancer metastasis and in hepatocellular carcinoma cell lines (HCC) producing hepatitis B virus (HBV) [157]. HULC exists as part of an intricate auto-regulatory network, which when perturbed, resulted in increased HULC expression (Figure 5a). The HULC RNA appeared to function as a ‘molecular decoy’ or ‘miRNA sponge’ sequestering miR-372, of which one function is the translational repression of PRKACB, a kinase targeting cAMP response element binding protein (CREB). Once activated, the CREB protein was able to promote HULC transcription by maintaining an open chromatin structure at the HULC promoter resulting in increased HULC transcription [158].

Another well known RNA that belongs to lncRNA subclass described in previous paragraph is HOX antisense intergenic RNA (HOTAIR) (see Figure 5b). HOTAIR is 2.2 kb gene localized within the human HOXC gene cluster on the long arm of chromosome 2. It has been shown that this lincRNA has a potential to regulate HOXD genes in trans via the recruitment of polycomb repressive complex 2 (PRC2), followed by the trimethylation of lysine 27 of histone H3 [7]. In general, the 5′ region of the RNA binds the PRC2 complex responsible for H3K27 methylation, while the 3′ region of HOTAIR binds LSD1 (flavin-dependent monoamine oxidase), a histone lysine demethylase that mediates enzymatic demethylation of H3K4Me2. HOTAIR exists in mammals, has poorly conserved sequences and considerably conserved structures, and has evolved faster than nearby HOXC genes [187]. HOTAIR was one of the first metastasis-associated lncRNAs, described to have a fundamental role in cancer. This lncRNA was found to be highly upregulated in both primary and metastatic breast tumors, showing up to 2000-fold increased transcription over normal breast tissue. This phenotype seems to be closely linked with PRC2-dependent gene repression induced by HOTAIR. High levels of HOTAIR expression correlate with both metastasis and poor survival rate, connecting lncRNAs with tumor invasiveness and patient prognosis [140]. In addition, it was observed that the high expression level of HOTAIR in hepatocellular carcinoma could be a candidate biomarker for predicting tumor recurrence in hepatocellular carcinoma patients who have undergone liver transplant therapy and might be a potential therapeutic target [188]. Huarte *et al*. [189] identified several lincRNAs that are regulated by p53. Furthermore, he proved that lincRNAs-p21 serves as a repressor in p53-dependent transcriptional responses, since inhibition of this lincRNA affected the expression of hundreds of gene targets enriched for genes normally repressed by p53.

While targeting cancer-specific miRNAs has proven to be successful, it will be necessary to design molecules with potential to inhibit lincRNAs. Gupta *et al*. [140] proved that these molecules can be depleted by siRNAs, but this possibility is quite complicated because of extensive secondary structures in lincRNAs [187]. Nevertheless, it is evident that cancer-associated lincRNAs may provide new approaches to the diagnosis and treatment of cancer.

### Long intronic non-coding RNAs

The biogenesis of long intronic ncRNAs is poorly understood at this time. Nevertheless, there are some indirect evidences that indicate an involvement of RNA polymerase II (RNAPII). Among such evidences belong a concordant and co-regulated expression profiles of many intronic ncRNAs and their corresponding protein-coding genes, the broad contribution of RNAPII associated transcription factors and physiological stimuli in the transcription of intronic ncRNAs as well the presence of poly(A+) tail [190-194]. Nonetheless, it is described that over 10% of long intronic poly(A+) ncRNAs are up-regulatated compared to only 4% of protein-coding transcripts after treatment with the RNAPII specific inhibitor α-amanitin [190,193,195]. These findings suggest that some intronic ncRNA and peculiar protein-coding RNAs could be transcribed by another RNA polymerase such as the recently described spRNAP-IV, whose transcriptional output seems to be enhanced by α-amanitin, or also could be transcribed by RNAP III [190,195-199].

Similarly to lincRNAs, there are also described evolutionary conserved long intronic ncRNAs sequences from mouse and human [200,201]. When the introns of a larger selection of vertebrates were aligned, the length of the conserved region became only 100 bp, while in the alignment of a smaller group of closely related species (human–mouse–cow–dog) the evolutionary conservation of the region extended to as much as 750 bp [201].

The widespread occurrence, tissue and subcellular expression specificity, evolutionary conservation, environment alteration responsiveness and aberrant expression in human cancers are features that accredit intronic ncRNAs to be mediators of gene expression regulation. A few sets of intronic ncRNAs have the same tissue expression pattern as the corresponding protein-coding genes, whereas others are inversely correlated. These findings point to complex regulatory relationships between intronic ncRNAs and their host loci [190,193,202,203]. Some small ncRNAs are encoded within intronic regions; moreover, intronic miRNAs tend to be present in large introns with 5′-biased position distribution, what correlates with the previous observation that most long intronic transcripts are expressed within first introns of the host genes. Thus, it is expected that a number of long intronic ncRNAs are processed into smaller ncRNAs [68,190,204,205]. Similar to lincRNAs HOTAIR, Heo *et al*. [206] described a long intronic noncoding RNA termed as cold assisted intronic non-coding RNA – COLDAIR, which is required for the vernalization-mediated epigenetic repression of FLC mediated by PRC2. Interestingly, the newest study of Tahira *et al*. [207] shows that long intronic non-coding RNAs are differentially expressed in primary and metastatic pancreatic cancer. Moreover, loci harbouring intronic lncRNAs differentially expressed in pancreatic ductal carcinoma metastases were enriched in genes associated to the MAPK pathway. These findings indicate potential relevance of this class of transcripts in biological processes related to malignant transformation and metastasis.

### Telomere-associated ncRNAs

Telomeres protect linear chromosome ends from being recognized and processed as double-strand breaks by DNA repair activities. This protective function of telomeres is essential for chromosome stability. Until recently, the heavily methylated state of subtelomeric regions, the gene-less nature of telomeres, and the observed telomere position effect led to the notion that telomeres are transcriptionally silent [208]. This hypothesis was recently challenged when several groups independently demonstrated that subtelomeric and telomeric regions, although devoid of genes, have the potential to be transcribed into telomeric UUAGGG-repeat containing ncRNAs (TERRA) [209-211]. TERRA molecules are conserved among eukaryotes and have been identified also in human. TERRA transcripts are synthesized from the C-rich strand and polyadenylated, and their synthesis is α-amanitin-sensitive, suggesting that they are transcripts of RNAPII [208,212]. TERRA molecules range between 100 bp and >9 kb in length and were reported to form intermolecular G-quadruplex structure with single-stranded telomeric DNA, but can also fold into a compact repeated structure containing G-quartets [211]. TERRA transcripts can be found throughout the different stages of the cell cycle, and their levels are affected by several factors that include telomere length, tumor stage, cellular stress, developmental stage, and telomeric chromatin structure [208].

TERRA most likely negatively regulates telomere length [211]. Increased TERRA levels by interfering with TERRA decay, such as the impairment of non-sense-mediated RNA decay in human cells or by deletion of the 5′–3′exonuclease Rat1p in Saccharomyces cerevisiae, are associated with a loss of telomere reserve [209,212]. Current models propose a role for TERRA in controlling telomerase activity. In yeast, the formation of a DNA/RNA hybrid between TERRA and telomeres is thought to inhibit elongation by telomerase, whereas in mammals, TERRA was shown to efficiently inhibit telomerase activity *in vitro*, presumably by base pairing with the template region of the RNA component of telomerase [208,210,212]. Caslini *et al*. [213] described that telomere uncapping through either TRF2 shelterin protein knockdown or exposure to telomere G-strand DNA oligonucleotides significantly increases the transcription of TERRA, an effect mediated by the functional cooperation between transcriptional regulator MLL and the tumor suppressor p53. Sampl *et al*. [214] found out that the expression of TERRA in patients with glioblastoma multiforme negatively correlates with the grade. Moreover, this finding of a diagnostic value of TERRA levels in astrocytoma WHO grade 2 to 4 corresponded with preliminary data in advances stages of human tumors of larynx, colon, and lymph node [210]. Unfortunately, it is largely unclear how the expression of TERRA and the amount of TERRA transcripts are regulated in the cell [208]. Nevertheless, TERRA opens new avenues for telomere research that will impact on telomere-associated diseases including many cancers [215].

### Long ncRNAs with dual functions

Until not long ago, ncRNAs were strictly considered as RNA molecules with regulatory functions but not associated with the protein coding capacity typical of messenger RNAs. However, the recent identification and characterization of bifunctional RNAs, i.e. RNAs for which coding capacity and activity as functional regulatory RNAs have been reported, suggests that a definite categorization of some RNA molecules is far from being straightforward [216]. The steroid receptor RNA activator (SRA) is a unique co-regulator that functions as a non-coding RNA, although incorporation of an additional 5′ region can result in translation of an SRA protein (SRAP) that also has co-activator activity [180,217,218]. SRA was initially shown to enhance gene expression through a ribonucleoprotein complex with steroid receptors and SRC-1 [217]. Currently, SRA is known as an RNA co-activator for many other nuclear receptors. In addition, SRA may act as an RNA scaffold for co-repressor complexes [216,219]. SRA transcripts have been identified in normal human tissues, with a higher expression in liver, skeletal muscle, adrenal and pituitary glands, whereas intermediate expression levels were observed in the placenta, lung, kidney and pancreas [217]. In some pathological cases, increased RNA levels of SRA were reported like in breast and ovarian tumors [179,220,221]. Interestingly, levels of SRA expression could be characteristic of tumor grade or particular subtypes of lesions among different tumors. Indeed, serous ovarian tumors showed higher levels of SRA than granulosa tumor cells [216,220].

### Pseudogene RNAs

Pseudogenes are gene copies that have lost the ability to code for a protein; they are typically identified through annotation of disabled, decayed or incomplete protein-coding sequences. These molecules have long been labeled as “junk” DNA, failed copies of genes that arise during the evolution of genomes. However, recent results showed that some pseudogenes appear to harbor the potential to regulate their protein-coding cousins [222,223]. Processed pseudogenes are made through retrotransposition of mRNAs, especially as a possible by-product of LINE-1 (Long INterspersed Elements) retrotransposition. Thus, these mRNAs are reverse transcribed and re-integrated into the genomic DNA [224,225]. The parent gene of the mRNA need not to be on the same chromosome as the retrotransposed copy. Retrotransposed mRNAs have three possible fates in the genome: formation of processed genes, formation of non-transcribed pseudogenes, or formation of pseudogenes transcribed into RNAs [222]. Interestingly, some of these RNAs exhibit a tissue-specific pattern of activation. Pseudogene transcripts can be processed into short interfering RNAs that regulate coding genes through the RNAi pathway. In another remarkable discovery, it has been shown that pseudogene RNAs are capable of regulating tumor suppressors and oncogenes by acting as microRNA decoys [223,225]. Moreover, Devor *et al*. [226] found out that primate-specific miRNAs, miR-220 and miR-492, each lie within a processed pseudogene. Several studies also show deregulated expression of these molecules during cancer progression, which provides evidence for the functional involvement of pseudogene RNAs in carcinogenesis and suggests these molecules as a potential novel diagnostic or therapeutic target in human cancers. One of these pseudogenes is myosin light chain kinase pseudogene (MYLK). MYLKP1 is partially duplicated from the original MYLK gene that encodes nonmuscle and smooth muscle myosin light chain kinase (smMLCK) isoforms and regulates cell contractility and cytokinesis. Despite strong homology with the smMLCK promoter (∼ 90%), the MYLKP1 promoter is minimally active in normal bronchial epithelial cells, but highly active in lung adenocarcinoma cells. Moreover, MYLKP1 and smMLCK exhibit negatively correlated transcriptional patterns in normal and cancer cells with MYLKP1 strongly expressed in cancer cells and smMLCK highly expressed in non-neoplastic cells. For instance, expression of smMLCK decreased in colon carcinoma tissues compared to normal colon tissues. Mechanistically, MYLKP1 overexpression inhibits smMLCK expression in cancer cells by decreasing RNA stability, leading to increased cell proliferation. These findings provide strong evidence for the functional involvement of pseudogenes in carcinogenesis and suggest MYLKP1 as a potential novel diagnostic or therapeutic target in human cancers [227]. Using massively parallel signature sequencing (MPSS) technology, RT-PCR, and 5′ rapid amplification of cDNA ends (RACE) a novel androgen regulated and transcribed pseudogene of kallikreins termed as KLK31P was discovered. It was further proved that this pseudogene may play an important role in prostate carcinogenesis [228]. He *et al*. [229] found out that pseudogene RNAs are also able to regulate a dosage of PTEN tumor suppressor during tumor development. Pseudogene RNAs however, warrant further investigation into the true extent of their function [223,227].

### Transcribed-ultraconserved regions

Ultraconcerved regions (UCRs) are a subset of conserved sequences that are located in both intra- and intergenic regions. They are 481 sequences, longer than 200 bp that are absolutely conserved between orthologous regions of human, rat, and mouse genomes [230]. Calin *et al*. [170] have proved in cancer systems that differentially expressed UCR could alter the functional characteristics of malignant cells. The link between genomic location of UCRs and analyzed cancer-related genomic elements is highly statistically significant and comparable to that reported for miRNAs. UCRs are frequently located at fragile sites and genomic regions involved in cancers. Using northern blot, qRT-PCR and microarray analysis, it was revealed that UCRs have distinct signatures in human leukemias and carcinomas [170].

Majority of UCRs are transcribed (T-UCRs) in normal human tissues, both ubiquitously and tissue specifically. From the molecular point of view, untranscribed UCRs might have regulatory functions as enhancers [231], while many functions can be assigned for T-UCRs, such as antisense inhibitors for protein-coding genes or other ncRNAs, including miRNAs. On the other hand, instead of T-UCRs interacting with protein-coding genes and miRNAs, it is possible that miRNAs control T-UCRs. Evidence supporting this predication is that many T-UCRs have significant antisense complementarity with particular miRNAs and negative correlation between expression of specific T-UCRs and predicted interactor miRNAs [170,232].

The expression of many T-UCRs is significantly altered in cancer, especially in adult chronic lymphocytic leukemias, colorectal and hepatocellular carcinomas and neuroblastomas [170]. Their aberrant transcription profiles can be used to distinguish types of human cancers and have been linked to patient outcome [233]. Especially in neuroblastoma, functional T-UCR annotations, inferred through a functional genomics approach and validated using cellular models, reveal associations with several cancer-related cellular processes such as apoptosis and differentiation [234]. Further, DNA hypomethylation induces release of T-UCR silencing in cancer cells. Studies of primary human tumors have shown that hypermethylation of T-UCR CpG islands is common event among the various tumor types. Thus in addition to miRNAs, another class of ncRNAs (T-UCRs) undergoes DNA methylation-associated inactivation in transformed cells, and so supports model that both epigenetic and genetic alterations in coding and noncoding sequences cooperate in human tumorigenesis. Most importantly, restoration of T-UCR expression was observed upon treatment with the DNA-demethylating agent [232]. Another study proved, that SNPs (single nucleotide polymorphisms) rs9572903 and rs2056116 in ultraconserved regions were associated with increased familial breast cancer risk [235]. Because of increasing number of studies concerning T-UCRs is published, it is supposed that the more specific roles of these molecules in cancer will be known in a short time.

## Conclusions and future perspectives

For a long time, the central dogma of molecular biology proposed RNA molecules primarily to be informational “messenger” between DNA and protein. But, surprisingly, only 2% of the human genome sequence encodes proteins, while a large part of it is devoted to the expression of ncRNAs, which are divided into two main groups according to their nucleotide length – small and long ncRNAs. These molecules are suggested to be important regulators of gene expression. Nevertheless, the two groups of ncRNAs are distinct in their biological functions and mechanisms of gene regulations. Small ncRNAs are involved mainly in the post-transcriptional gene regulation using translational repression or RNAi pathway, while long ncRNAs are much more involved in epigenetic regulation. In many cases, differential expression of ncRNAs is becoming recognized as a one of the hallmarks of cancer cell, indicating their potential usage as the novel diagnostic, prognostic, or predictive biomarkers. Growing evidence also suggests that ncRNAs have the promising potential in targeted regulation of gene expression and, therefore, in cancer targeted therapy. However, the function of many ncRNAs remains unknown and it will be necessary to discover the precise mechanisms by which are these molecules involved in carcinogenesis.

## Competing interest

The authors declare that they have no competing interests.

## Authors’ contributions

SJ and FP drafted the manuscript, SM and SO revised the manuscript critically for important and intellectual content. All authors read and approved the final manuscript. All authors read and approved the final manuscript.
